# The Commonality and Individuality of Human Brains When Performing Tasks

**DOI:** 10.3390/brainsci14020125

**Published:** 2024-01-25

**Authors:** Jie Huang

**Affiliations:** Department of Radiology, Michigan State University, East Lansing, MI 48824, USA; huangj@msu.edu; Tel.: +1-517-884-3246

**Keywords:** human brain function, whole brain activity, individuality

## Abstract

It is imperative to study individual brain functioning toward understanding the neural bases responsible for individual behavioral and clinical traits. The complex and dynamic brain activity varies from area to area and from time to time across the entire brain, and BOLD-fMRI measures this spatiotemporal activity at large-scale systems level. We present a novel method to investigate task-evoked whole brain activity that varies not only from person to person but also from task trial to trial within each task type, offering a means of characterizing the individuality of human brains when performing tasks. For each task trial, the temporal correlation of task-evoked ideal time signal with the time signal of every point in the brain yields a full spatial map that characterizes the whole brain’s functional co-activity (FC) relative to the task-evoked ideal response. For any two task trials, regardless of whether they are the same task or not, the spatial correlation of their corresponding two FC maps over the entire brain quantifies the similarity between these two maps, offering a means of investigating the variation in the whole brain activity trial to trial. The results demonstrated a substantially varied whole brain activity from trial to trial for each task category. The degree of this variation was task type-dependent and varied from subject to subject, showing a remarkable individuality of human brains when performing tasks. It demonstrates the potential of using the presented method to investigate the relationship of the whole brain activity with individual behavioral and clinical traits.

## 1. Introduction

Our perception, cognition and action are mediated by brain function. Brain functional organization consists of multiple functional systems from simple systems such as sensorimotor and visual systems to complex cognitive systems such as language. These separated systems seem to suggest a functional segregation of brain function, but human behavior is the result of an integrated functioning of the whole brain’s activity. For example, performing a simple visually cued finger-tapping (FT) task evokes both visual and sensorimotor systems. The information of visual cue is first processed through the visual system and then triggers the motor system to plan and execute the task of tapping fingers, which consequently evoke the somatosensory system. This performance may vary from trial to trial due to possible attention variation and interaction among these systems, resulting in a task-evoked whole brain activity that may vary from trial to trial. Investigating this trial-by-trial whole brain activity may provide insight to understand the relationship of brain activity with individual behavior.

Even without performing any specific task, human brain intrinsic activity accounts for 20% of all the energy consumed by the body to maintain the operations of the brain, and these operations involve the acquisition and maintenance of information for interpreting, responding to and predicting environmental demands [1]. This intrinsic activity, i.e., the resting-state (rs) activity, is spontaneous but exhibits a surprising level of spatial and temporal organization across the entire brain [2,3]. Numerous rs-fMRI studies demonstrate the existence of multiple functional connectivity networks (FCNs) within the entire brain [4,5,6]. The sliding window approach of analyzing rs-fMRI data reveals the time-varying behavior of these FCNs, demonstrating dynamic FCN from time to time [7]. These rs-fMRI studies, however, are group-based studies that analyze the fMRI data in a standard template space. It remains to be explored how to extend the rs-fMRI technique to investigate the relationship of window-by-window activity of FCNs with individual behavior.

A recent combined rs- and task-fMRI study revealed the relationship of functional connectivity of the sensorimotor and visual cortical networks between resting and task states [8]. The study showed that the intrinsic and task-evoked FCNs shared a common network and the task enhanced the coactivity within that common network in comparison to the intrinsic activity. However, the task activated only partial but not whole of the intrinsic FCN. The task also activated substantially additional areas outside the intrinsic FCN, demonstrating the different functioning of the intrinsic FCNs compared to the task-evoked FCNs. Another combined rs- and task-fMRI study compared the intrinsic activity with the activity evoked by tasks at several levels of analysis from an FT-activated area within the primary sensorimotor cortex to the entire brain [9]. Contrary to our intuition, the intrinsic activity was found to be substantially larger than the task activity at all levels of analysis. The study also found that, for the task state, the brain controlled the intrinsic activity not only during the task period but also during the rest between tasks. The brain activated a task-specific network only when the task was performed but kept it relatively “silent” for other different tasks, and at the same time, it simultaneously controlled the activation of all task-specific networks during the performance of each task. These results demonstrate a dynamic whole brain activity that may depend on each individual brain’s controlling of its activity, and consequently, the whole brain activity may vary from task trial to trial for each individual brain. Accordingly, investigating this trial-by-trial whole brain activity may provide insight to understand the relationship of brain activity with individual behavior.

The complex and dynamic brain activity varies from area to area and from time to time across the entire brain. BOLD-fMRI measures this spatiotemporal activity at large-scale systems level [10,11]. Numerous fMRI studies have demonstrated its effectiveness and reliability in investigating the common features of human brain functional organization at a group level (i.e., the commonality across the subjects within a group) and the effects of brain disorders on brain activity. It is imperative, however, to study individual brain functioning for understanding the neural bases responsible for individual behavioral and clinical traits. Person-specific neuroimaging approaches in investigating individual brain functioning have been reported in the literature [12,13,14,15]. In this study we present a novel method to investigate task-evoked whole brain activity that varies not only from person to person but also from task trial to trial within each task type, offering a means of characterizing the individuality of human brains when performing tasks.

## 2. Materials and Methods

We extend our previous four studies [9,16,17,18]. This study analyzed the same fMRI data. It used the same subjects, same image acquisition, and similar image preprocessing procedures. We briefly describe each paragraph. For more information, refer to our previous study [16].

Participants: 9 healthy subjects (4 female and 5 male, ages 21–55 years old) participated in the study.

Image acquisition: functional brain images were acquired on a GE 3.0 T clinical scanner with an 8-channel head coil using a gradient echo Echo-Planar-Imaging pulse sequence (TE/TR = 28/2500 ms, flip angle 80°, FOV 224 mm, matrix 64 × 64, slice thickness 3.5 mm, and spacing 0.0 mm). Thirty-eight axial slices to cover the whole brain were scanned, and the first three volume images were discarded. Each subject undertook a 12 min task-fMRI scan while performing three different tasks. Each task was presented eight times, for a total of 24 task trials, and the task presentation was interleaved. Each trial comprised a 6 s task period followed by a 24 s rest period, resulting in 12 volume images for each task trial. Task 1 was a word-reading (WR) paradigm: subjects silently read English words. Task 2 was a pattern-viewing (PV) paradigm: subjects viewed a black-and-white striped pattern with a spatial frequency of 2.8 cycles per degree. Task 3 was a visually cued FT paradigm: each subject tapped the five fingers of their right hand as quickly as possible in a random order. During the 24 s rest period, subjects were asked to focus their eyes on a small fixation mark at the screen center and try not to think of anything. After the task-fMRI scan, T1-weighted whole brain MR images were also acquired using a 3D IR-SPGR pulse sequence.

Image preprocessing: image preprocessing of the functional images was performed using AFNI (analysis of functional neuro images) software (http://afni.nimh.nih.gov/afni, accessed on 11 December 2023, Version AFNI_2011_12_21_1014) [16,19]. It included removing spikes, slice-timing correction, motion correction, spatial filtering with a Gaussian kernel with a full-width half-maximum of 4.0 mm, computing the mean volume image, bandpassing the signal intensity time courses to the range of 0.009–0.08 Hz, and computing the relative signal change (%) of the bandpassed signal intensity time courses. After these preprocessing steps, further image analysis was carried out using in-house developed Matlab-based software (MATLAB R2019b) algorithms.

Quantification of trial-by-trial brain activity within each subject: task-evoked brain activity can be characterized by an ideal BOLD response time signal [20]. This ideal response was generated by convolving the 6 s task on and 24 s task off temporal paradigm with a hemodynamic response function, using the 3dDeconvolve program in AFNI with the convolution kernel SPMG3. For each task trial, the temporal correlation (TC) r of this ideal time signal with the time signal of every point in the brain yields a full spatial map that characterizes the whole brain’s functional co-activity (FC) relative to the task-evoked ideal response. This computation results in 8 FC maps for each of the three task categories and each subject. A given task should evoke similar FC maps by repeating the task. For any two task trials, regardless of whether they are the same task or not, the spatial correlation (SC) R of their corresponding two FC maps over the entire brain quantifies the similarity between these two maps, offering a means of investigating the variation in the whole brain activity trial to trial. For each individual subject, the SC R values of all pairwise FC maps for all task trials measure the variations in these FC maps and therefore quantify the individuality of that subject in performing these tasks.

For each subject, each FC map uniquely characterizes the whole brain’s activity when performing a given task trial for that subject, offering a marker to distinguish tasks based on their FC maps. To test this prediction, we choose one FC map from each task category and use these three FC maps as their corresponding task markers to predict the task type of each trial for the remaining 21 trials. For a given test trial, the predicted task type is the one with the largest SC R among the three chosen FC maps. There are a total of 512 combinations in choosing three FC maps from the three task categories and 21 test trials for each choice, resulting in a total of 10,752 tests for each individual subject. The correct rate of identifying these task trials further quantifies the individuality of that subject in performing these tasks.

The commonality of brain activity across the subjects: To examine this commonality, for each subject, we first compute the task-mean FC map averaged over the 8 FC maps for each task category and use this task-mean FC map to represent the whole brain’s FC in performing that task. Then, using AFNI, we convert all 27 task-mean FC maps from the 9 subjects to a standard template space (icbm452) for group analysis. In this standard template space, for each task category, the SC R of any two paired FC maps over the entire brain measures the similarity of brain activity between the corresponding two subjects in performing that task, offering a means of quantifying the commonality of brain activity across the subjects in performing these tasks.

## 3. Results

**Figure 1:**
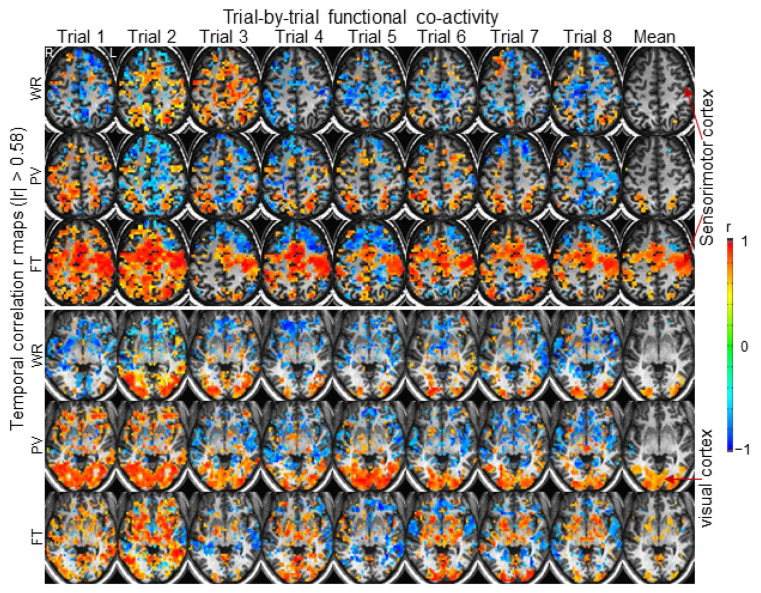
Illustration of the variation in trial-by-trial whole brain FC relative to the task-evoked ideal response for a representative subject. For the illustration purpose, these TC r maps were presented with threshold |r| > 0.58 (N = 12, *p* < 0.05). The right-hand FT-evoked activity in both the left sensorimotor cortex and supplementary motor area was consistent for all 8 trials, but the FC for other cortical areas within the selected slice varied substantially from trial to trial (the third row in the top panel). Similarly, the PV-evoked activity in the visual cortex was also consistent for all 8 trials, though its degree varied substantially from trial to trial (the second row in the bottom panel). WR: word reading; PV: pattern viewing; FT: finger tapping; R: right; L: left.

**Figure 2:**
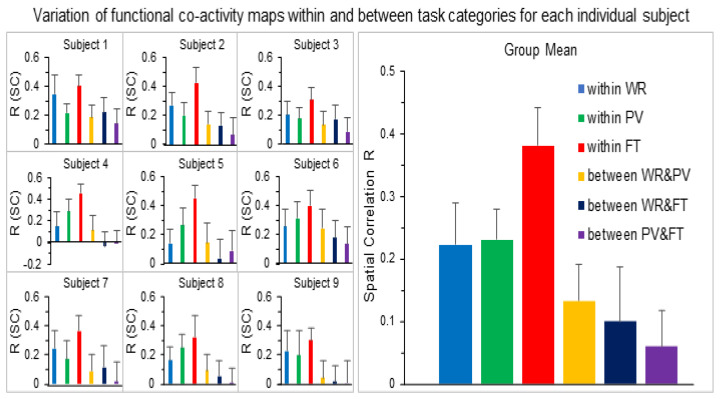
Comparison of the mean spatial correlation (SC) R of pairwise FC maps within each task category and between two task categories for each individual subject (left three columns) and the group-mean values averaged over the nine subjects (right plot). WR: word reading; PV: pattern viewing; FT: finger tapping. The error bars indicate the standard deviations.

For each subject, based on the T1 and EPI images, in the original MRI space, we generated a mask to cover the entire brain. For each subject and each task trial, we computed the TC r of the ideal BOLD response with the time signal of every voxel within the brain mask to yield the FC map for that subject and that task. The trial-by-trial variation in this TC r map across the brain for a representative subject is illustrated in Figure 1. Then, for each subject, we computed the SC R for (1) all pairwise FC maps within each task category (a total of 28 paired FC maps for each task category) and (2) all pairwise FC maps between any two task categories (a total of 64 paired FC maps between two task categories) (Table 1). The mean R within the FT category had the largest value among all categories for each individual subject, showing the greatest similarity of the whole brain’s activity when performing the FT task (Figure 2). The mean R within each of the other two tasks (WR and PV) was substantially reduced for every subject, demonstrating that the whole brain’s activity varied substantially from trial to trial when performing these tasks. The mean R of paired FC maps between two task categories was relatively smaller in comparison to that within a task category and varied substantially from subject to subject, consistent with the expectation that the difference in the brain’s activity of performing two different tasks should be larger than that of performing the same task repeatedly.

**Figure 3:**
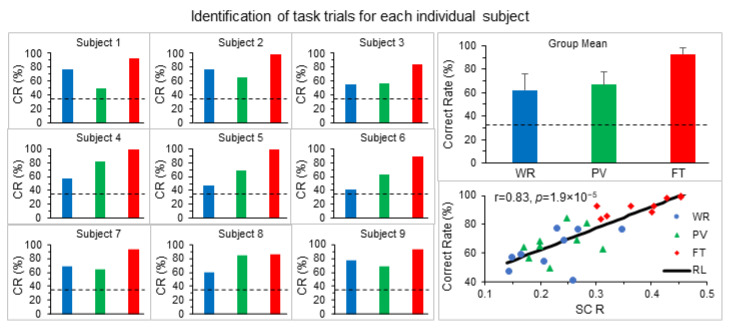
The correct rate (CR) of identifying task trials based on their FC maps for each individual subject (left three columns) and the group mean averaged over the nine subjects (right top plot). The dash lines indicate the random selection CR of 33.3% (1 out of 3 choices). The right bottom plot illustrates the association of the correct rate with the SC R across the subjects. WR: word reading; PV: pattern viewing; FT: finger tapping; RL: regression line. The error bars indicate the standard deviations.

Each FC map uniquely characterized the whole brain’s activity in performing that task trial for that subject. Using FC map as a marker to distinguish tasks based on their FC maps, for each individual subject, the correct rate of identifying these task trials was higher than that of random selection correct rate of 33.3% for each of the three task categories (Figure 3). This correct rate was substantially and consistently higher for the FT task than that for the other two tasks of WR and PV, independent of the subjects. For all subjects, the correct rate of identifying these task trials ranged from 41.2% to 77.4% with a mean of 62.3 ± 13.4% (SD) for the WR trials, 50.0% to 84.5% with a mean of 66.9 ± 10.9% for the PV trials and 83.9% to 99.8% with mean 92.7 ± 5.8% for the FT trials, respectively. A paired t-test analysis showed that this correct rate was significantly larger than that of random selection for each task category (largest *p* < 0.0002). For each subject, we computed the mean SC R within each task category and investigated the association of the correct rate of identifying that task with this mean SC R value. A significant correlation between the R and correct rate was observed across all subjects (r = 0.83, *p* = 1.9 × 10^−5^ for N = 27) (Figure 3).

**Figure 4:**
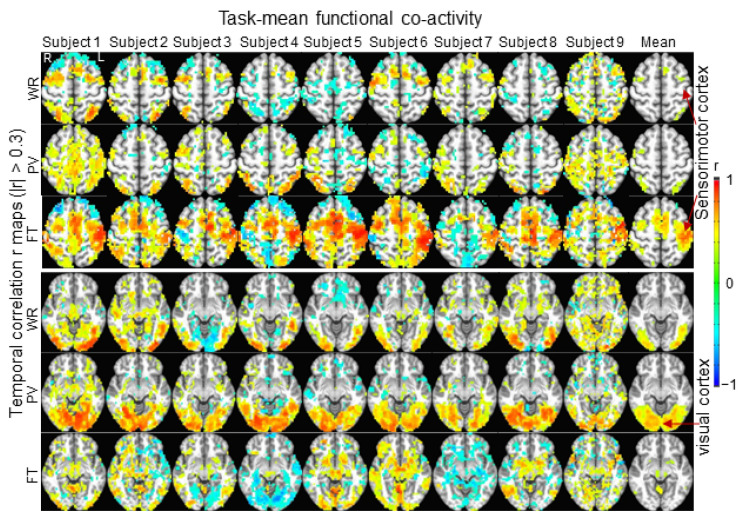
Illustration of the whole brain task-mean FC for each of the three task categories and each subject. For the illustration purpose, these task-mean TC r maps were presented with threshold |r| > 0.3. The right-hand FT-evoked activity in both the left sensorimotor cortex and supplementary motor area was consistent for all 9 subjects, though its degree varied substantially from subject to subject (the third row in the top panel). Similarly, the PV-evoked activity in the visual cortex was also consistent for all 9 subjects (the second row in the bottom panel). This whole brain task-mean FC showed a large variation not only between different task types but also from subject to subject. WR: word reading; PV: pattern viewing; FT: finger tapping; R: right; L: left.

**Figure 5:**
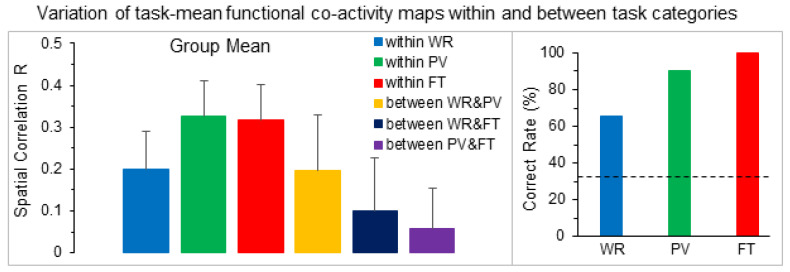
Comparison of the group-mean spatial correlation (SC) R of pairwise task-mean FC maps within each task category and between two task categories averaged over all subjects (left plot). The correct rate of identifying tasks using each subject’s three task-mean FC maps as the three task markers to identify the task for the rest eight subjects (right plot). The dash lines indicate the random selection CR of 33.3% (1 out of 3 choices). WR: word reading; PV: pattern viewing; FT: finger tapping. The error bars indicate the standard deviations.

The task-mean FC map for each task category and each subject is illustrated in Figure 4. This task-mean FC map showed a substantial variation not only between different tasks but also from subject to subject (Figure 5). The group-mean SC R was larger within each task category than that between task categories, showing the commonality of these task-evoked FC maps across all subjects. Using each subject’s three task-mean FC maps as the three task markers to identify the 24 tasks for the rest eight subjects, the correct rate of task identification was 65.3% for WR, 90.3% for PV and 100% for FT, respectively, substantially higher than the correct rate of 33.3% for random selection.

## 4. Discussion and Conclusions

This study examined trial-by-trial whole brain activity for each individual subject. As expected, the right-hand FT task activated the left sensorimotor cortex and supplementary motor area consistently across all eight trials for each individual subject. The size of this activation in these areas, however, varied not only from trial to trial (Figure 1) but also from subject to subject (Figure 4). Outside the sensorimotor system, the cortical activity varied substantially from trial to trial for all subjects; i.e., some areas showed a positive activity relative to the FT-evoked ideal response for one trial but a negative activity for another trial, demonstrating a varied whole brain activity when performing these repeated FT tasks. This variation in the whole brain activity from trial to trial characterizes the individuality of the human brains in performing these repeated FT tasks. Similar results were observed for the other two tasks of WR and PV with the same conclusion (Figure 1 and Figure 4). The SC R of two FC r maps quantifies the degree of the similarity of the whole brain activity in performing the two tasks, regardless of whether they are the same task or not; i.e., the larger the R value, the smaller the variation in the whole brain activity. Although the FT task showed the smallest variation consistently across all nine subjects, this variation varied substantially from trial to trial for every subject as reflected in their corresponding large values of standard deviation (Figure 2), providing evidence to show the individuality of the human brains in performing these repeated FT tasks. In comparison to the FT task, the WR and PV tasks showed larger variations that also varied from subject to subject, providing further evidence to demonstrate the individuality of the human brains in performing these tasks.

For a given task, the FC r map across the entire brain reflects the whole brain’s activity in performing that task and therefore provides a marker to identify the task based on its FC r map. The correct rate of identifying these tasks was higher than that of random selection for each individual subject (Figure 3), and as a group, this correct rate was significantly higher than that of random selection for each task category (largest *p* < 0.0002). As the SC R of any two FC r maps over the entire brain measures the similarity between these two maps and the correct rate of identifying these two tasks depends on this similarity, as expected, the correct rate of identifying these tasks was found to be positively associated with the SC R (*p* = 1.9 × 10^−5^) (Figure 3). Among the three tasks of WR, PV and FT, the FT task had the largest R value followed by PV and then WR, indicating a possible inverse relationship of this R value with the degree of simplicity of the task. These results demonstrate the potential of using the presented method to investigate the relationship of the whole brain activity with individual behavioral and clinical traits.

For each subject, the task-mean FC r map of each task category reflects the common brain activity in performing that task. This mean FC map substantially reduced the trial-by-trial variation in the whole brain activity (last column in Figure 1). However, it varied substantially from subject to subject for each of the three tasks (Figure 4), showing a large variation in the whole brain activity from subject to subject when performing the same task. This large variation was also reflected in the large value of standard deviation of the SC R values for both within each task category and between any two task categories (left plot in Figure 5), demonstrating a limited commonality of the whole brain activity across these subjects when performing the same task. It provides further evidence to demonstrate the imperative of developing novel method to investigate the relationship of the whole brain activity with individual behavioral and clinical traits.

It may be worth comparing our presented method with those approaches reported in the literature in regard to the person-specific neuroimaging approach for studying individual brain functioning and its relationship to personal traits. First, to the best of our knowledge, we have not seen any method that can measure trial-by-trial whole brain activity within each task category for each individual subject. Second, most task-fMRI studies comprise group-based analysis aiming to identify regions of common activation or common functional networks across participants. Such an analysis requires the transference of each individual data to a standard template and then their analysis of as a group. This approach is effective and reliable in identifying the commonality of brain functional organization across a group as demonstrated by numerous fMRI studies. However, it may ignore important differences across the participants that might be responsible for individual traits. This is because individual brains may differ in size and shape, functional areas may vary in anatomical location across individuals, and abnormal brain structure may be associated with neurological disorders [21,22,23,24,25]. Accordingly, it is imperative to be able to analyze the fMRI data for each individual subject. Third, although person-specific approaches can effectively and reliably identify individual from group, they use either a pre-defined functional brain atlas or group-based parcellations and/or functional networks defined in a standard template space as their frameworks to carry out their analyses [12,26,27,28,29,30]. In comparison, the analysis of our presented method is conducted in the original MRI space for each individual participant, which might be crucial for applying the BOLD-fMRI technique to daily clinical practice.

In clinical practice, fMRI has been applied to presurgical planning and preoperative risk assessment for brain tumor and epilepsy surgeries [31,32,33]. To preserve language from surgical damage is a challenging but crucial task. It requires a precise mapping of language areas and associated networks. One main challenge is to dissociate task-associated from language-essential neural activity with fMRI because even a simple task may evoke multiple networks. One language fMRI mapping study with direct cortical stimulation of 40 consecutive patients with gliomas showed a 37.1% sensitivity and 83.4% specificity of the fMRI mapping in identifying the language areas, demonstrating the demand of substantially improved language fMRI mapping in clinical practice [34]. This result is consistent with our observed large variation in the WR task in the whole brain activity for both within each subject (Figure 1 and Figure 2) and between subjects (Figure 4 and Figure 5). As our presented method enables us to analyze trial-by-trial whole brain activity for each individual subject, it may improve the precision of language fMRI mapping. Furthermore, combining artificial intelligence with this method may develop a more effective and reliable method to examine individual human brain functioning that is crucial in daily clinical practice.

## 5. Conclusions

This study presented a novel method to examine trial-by-trial whole brain activity for each individual subject, providing insights for investigating the individuality of human brains when performing tasks. The results demonstrated a substantially varied whole brain activity from trial to trial for each task category. The degree of this variation was task type-dependent and varied from subject to subject, showing a remarkable individuality of human brains when performing tasks. It demonstrates the potential of using the presented method to investigate the relationship of the whole brain activity with individual behavioral and clinical traits.

## Figures and Tables

**Table 1:** Similarity of trial-by-trial whole brain FC within each task category and between any two task categories for each individual subject. This similarity was measured with the SC R of pairwise FC maps over the brain mask. The number of voxels within the brain mask varied from subject to subject with a mean brain size of 1085 ± 96 cm^3^. WR: word reading; PV: pattern viewing; FT: finger tapping; Min: minimum; Max: maximum; MN: mean; SD: standard deviation.

**Subject**	**Number of Voxels**	**R within WR Category**				**R within PV Category**				**R within FT Category**			
		**Min**	**Max**	**MN**	**SD**	**Min**	**Max**	**MN**	**SD**	**Min**	**Max**	**MN**	**SD**
1	23,542	0.12	0.55	0.35	0.13	0.11	0.36	0.22	0.07	0.25	0.53	0.41	0.07
2	27,035	0.10	0.44	0.27	0.09	0.05	0.36	0.20	0.09	0.19	0.57	0.43	0.10
3	29,249	0.03	0.40	0.21	0.09	0.04	0.29	0.18	0.07	0.15	0.42	0.31	0.08
4	22,005	−0.05	0.41	0.15	0.13	0.05	0.48	0.28	0.11	0.20	0.55	0.45	0.09
5	25,877	−0.08	0.31	0.14	0.10	−0.04	0.47	0.27	0.12	0.30	0.63	0.45	0.09
6	23,951	0.06	0.52	0.26	0.12	0.06	0.55	0.31	0.12	0.13	0.64	0.40	0.11
7	23,681	0.00	0.47	0.24	0.13	−0.06	0.41	0.17	0.13	0.12	0.61	0.36	0.11
8	26,840	0.01	0.40	0.16	0.09	0.05	0.42	0.25	0.09	0.05	0.59	0.32	0.15
9	25,528	−0.01	0.48	0.23	0.14	−0.10	0.54	0.20	0.17	0.16	0.47	0.30	0.09
MN	25,301	0.02	0.44	0.22	0.11	0.02	0.43	0.23	0.11	0.17	0.56	0.38	0.10
SD	2229	0.06	0.07	0.07	0.02	0.07	0.09	0.05	0.03	0.07	0.07	0.06	0.02
**Subject**	**Number of voxels**	**R between WR and PV**				**R between WR and FT**				**R between PV and FT**			
		**Min**	**Max**	**MN**	**SD**	**Min**	**Max**	**MN**	**SD**	**Min**	**Max**	**MN**	**SD**
1	23,542	−0.03	0.36	0.19	0.09	0.01	0.43	0.22	0.10	−0.05	0.41	0.14	0.10
2	27,035	−0.07	0.35	0.14	0.09	−0.10	0.27	0.13	0.09	−0.27	0.28	0.06	0.12
3	29,249	−0.09	0.43	0.14	0.09	−0.00	0.35	0.18	0.10	−0.10	0.29	0.09	0.10
4	22,005	−0.20	0.43	0.11	0.13	−0.28	0.25	−0.04	0.14	−0.22	0.21	−0.00	0.12
5	25,877	−0.16	0.43	0.15	0.14	−0.26	0.34	0.04	0.13	−0.22	0.39	0.09	0.14
6	23,951	−0.07	0.56	0.25	0.13	−0.06	0.49	0.18	0.12	−0.10	0.35	0.14	0.12
7	23,681	−0.16	0.36	0.09	0.11	−0.27	0.47	0.12	0.15	−0.28	0.34	0.02	0.13
8	26,840	−0.18	0.33	0.10	0.11	−0.20	0.27	0.06	0.10	−0.23	0.23	0.00	0.10
9	25,528	−0.23	0.32	0.04	0.12	−0.18	0.38	0.02	0.11	−0.39	0.35	−0.00	0.16
MN	25,301	−0.13	0.40	0.13	0.11	−0.15	0.36	0.10	0.12	−0.20	0.32	0.06	0.12
SD	2229	0.07	0.08	0.06	0.02	0.11	0.09	0.09	0.02	0.11	0.07	0.06	0.02

## Data Availability

Data are available on request due to restrictions of protecting the privacy of the research data. The data presented in this study are available on request from the corresponding author. The data are not publicly available as they are stored on a publicly inaccessible hard drive.
